# Hypoxic and hypercapnic burrow conditions lead to downregulation of free triiodothyronine and hematocrit in Ansell’s mole-rats (*Fukomys anselli*)

**DOI:** 10.1007/s00360-023-01526-0

**Published:** 2023-12-07

**Authors:** Yoshiyuki Henning, Kamilla Adam, Patricia Gerhardt, Sabine Begall

**Affiliations:** 1 Institute of Physiology, University Hospital Essen, University of Duisburg-Essen, Hufelandstr. 55, 45147 Essen, Germany; 2 Department of General Zoology, Faculty of Biology, University of Duisburg-Essen, Essen, Germany

**Keywords:** Thyroid hormones, Erythropoiesis, African mole-rat, Ecophysiology, Hypoxia, Hypercapnia

## Abstract

African mole-rats live in self-dug burrow systems under hypoxic and hypercapnic conditions. Adaptations to hypoxia include suppression of resting metabolic rate (RMR) and core body temperature (*T*_b_). Because the thyroid hormones (THs) thyroxine (T4) and triiodothyronine (T3) are positive regulators of RMR and *T*_b_, we hypothesized that serum TH concentrations would also be downregulated under hypoxic conditions. To test this hypothesis, we kept Ansell’s mole-rats (*Fukomys anselli*) in terraria filled with soil in which they were allowed to construct underground burrows to achieve chronic intermittent hypoxia and hypercapnia. The animals stayed in these hypoxic and hypercapnic burrows voluntarily, although given the choice to stay aboveground. We collected blood samples before and after treatment to measure serum T4 and T3 concentrations as well as hematological parameters. The free fraction of the transcriptionally-active T3 was significantly decreased after treatment, indicating that cellular TH signaling was downregulated via peripheral mechanisms, consistent with the assumption that aerobic metabolism is downregulated under hypoxic conditions. Furthermore, we found that hematocrit and hemoglobin concentrations were also downregulated after treatment, suggesting that oxygen demand decreases under hypoxia, presumably due to the metabolic shift towards anaerobic metabolism. Taken together, we have identified a potential upstream regulator of physiological adaptations to hypoxia in these highly hypoxia-tolerant animals.

## Introduction

The subterranean niche is home to at least 250 rodent species worldwide (Begall et al. 2007). Life in self-dug underground burrows provides advantages, such as protection from predators and stable ambient temperatures. On the other hand, subterranean environmental conditions can be harsh, encompassing low food availability, high humidity, or constant darkness. Another major factor that makes the subterranean lifestyle challenging is the atmosphere. There are controversial reports about the O_2_ availability in underground burrows, ranging from hypoxic and hypercapnic conditions to an atmosphere that does not significantly deviate from aboveground conditions (Shams et al. 2005, Holtze et al. 2018, Burda et al. 2007, McNab 1966, Braude et al. 2021). These diverging results might be explained by methodological differences, but it was also reported that, e.g., soil composition or rainfall affects gas exchange and thereby also the O_2_ and CO_2_ concentrations (Arieli 1979). Nevertheless, many subterranean rodents were reported to exhibit pronounced hypoxia and hypercapnia tolerance as compared to the aboveground rodents, indicating that hypoxic and hypercapnic conditions are regularly faced in underground burrows (Shams et al. 2004, 2005; Ivy et al. 2020; Liu et al. 2010; Zhang and Pamenter 2019a, b; Park et al. 2017; Arieli and Ar 1979; Schmidt et al. 2017). In African mole-rats (family: Bathyergidae), hypoxia tolerance is quite-well characterized. The naked mole-rat (*Heterocephalus glaber*) is often referred to as the most hypoxia-tolerant species among African mole-rats. As such, these animals can tolerate acute hypoxia of 3–7% O_2_ for several hours (Nathaniel et al. 2012; Cheng et al. 2021b), chronic hypoxia of 8–11% O_2_ for several days (Chung et al. 2016; Cheng et al. 2021a; Farhat et al. 2020) and even survive anoxia for up to 18 min (Park et al. 2017). Tolerance to hypercapnia is also highly pronounced, as these animals survive exposure to 80% CO_2_ for 5 h (Park et al. 2017). The recent reports have revealed that other bathyergid mole-rats also tolerate hypoxia and hypercapnia in a similar extent as naked mole-rats (Ivy et al. 2020; Zhang and Pamenter 2019a, b; Logan et al. 2020; Devereaux et al. 2023), pointing out that this remarkable hypoxia/hypercapnia tolerance is a common trait in bathyergid mole-rats. Moreover, hypoxia tolerance is also highly pronounced in phylogenetically distant species, such as blind mole-rats (*Spalax* sp.) (Shams et al. 2004; Avivi et al. 2005) and the golden-mantled ground squirrel (*Spermophilus lateralis*) (Barros et al. 2001), suggesting that this trait has evolved convergently in fossorial/subterranean rodents.

Two major physiological mechanisms to adapt to hypoxic conditions in mammals are downregulation of core body temperature (*T*_b_) and resting metabolic rate (RMR) (Frappell et al. 1992; Barros et al. 2001). In bathyergid mole-rats, RMR and *T*_b_ are naturally low (Šumbera 2019; Zelová et al. 2007; Schielke et al. 2017) and decrease even more under hypoxic conditions. In several bathyergid species, RMR and *T*_b_ were reported to decrease under hypoxic conditions by approximately 50% and 2–6 °C, respectively (Ivy et al. 2020; Cheng et al. 2021b; Kirby et al. 2018; Pamenter et al. 2019), but the upstream mechanism of this hypoxia response has not been identified thus far.

Previously, we have characterized the thyroid hormone (TH) system of two bathyergid mole-rat species, the Ansell’s mole-rat (*Fukomys anselli*) and the naked mole-rat. THs circulating in the blood are mainly thyroxine (T4) and triiodothyronine (T3), from which T4 serves mainly as a prohormone and T3 is the transcriptionally-active form binding to nuclear TH receptors. In the blood, > 99% of TH is bound to transport proteins, and only the remaining free fractions of T4 and T3 (fT4; fT3) can enter target cells to exert their function. The combined bound and free fractions of T4 and T3 are referred to as TT4 and TT3, respectively (van der Spek et al. 2017). There are strong indications that the TH system of Ansell’s mole-rats and naked mole-rats is involved in physiological adaptations to the subterranean habitat (Gerhardt et al. 2023; Henning et al. 2014; Buffenstein et al. 2001): as such, these animals have very low serum fT4 and TT4 concentrations, which results in a hypothyroid phenotype in terms of RMR and *T*_b_, because THs are positive regulators of these traits by, e.g., increasing energy expenditure or thermogenesis (Mullur et al. 2014; de Jesus et al. 2001). Therefore, we predicted that the TH system of mole-rats also contribute to the metabolic and thermoregulatory adaptations to hypoxic conditions. To test this prediction, we kept Ansell’s mole-rats in a semi-natural environment. In their natural, subterranean habitat, Ansell’s mole-rats live in families of up to 13 individuals with one breeding pair. They spend most of their lives underground and are rarely seen aboveground. Despite their small body mass of about 100 g, they build large burrow systems consisting of tunnels up to 2.8 km in length, which are located between a few centimeters and 2 m below ground. Nest chambers are usually found at approximately 50 cm depth (Šklíba et al. 2012). To simulate these environmental conditions in our experimental setting, we housed the animals in a glass terrarium filled with organic soil in which they could build underground burrows. These burrows were maintained hypoxic and hypercapnic for several days and we measured serum TH concentrations before and after the treatment period. As an additional parameter, we conducted a complete blood count to determine erythrocyte properties to examine whether oxygen transport properties are affected by the treatment and whether a potential functional relationship between changes in TH concentrations and erythrocyte properties can be inferred. We hypothesized that TH concentrations are downregulated and erythropoiesis is upregulated upon chronic hypoxia and hypercapnia.

## Materials and methods

### Animal husbandry

Ansell’s mole-rats were kept at the animal facility of the Department of General Zoology of the University of Duisburg-Essen. All animals were housed as family groups in glass terraria on wood shavings and fed ad libitum with carrots and potatoes (three times per week) as well as apples, grain, and lettuce (once a week). Room temperature and humidity were kept constant at 24 ± 1 °C and 50 ± 3%, respectively. Light conditions were 12D:12L. All animal experiments were conducted in accordance with the German Regulations for Laboratory Animal Science (GV-SOLAS) and were approved by the North Rhine-Westphalia State Environment Agency (LANUV, permit number: 81-02.04.2019.A455).

### Intermittent hypoxia/hypercapnia treatment

The main aim was to achieve intermittent hypoxia/hypercapnia in a semi-natural environment. For this purpose, 12 animals originating from three families (Table 1) were housed in groups of 3–5 individuals in a glass terrarium (80 × 35 × 40 cm) filled with organic soil. The terrarium was closed with a meshed iron lid equipped with vents to ensure proper ventilation and feeding. We left 5–8 cm of space between the lid and the soil to allow access to ambient air to simulate field conditions. Four holes along the vertical axis of the terrarium were used to measure gas concentrations at different depths (5 cm, 15 cm, 25 cm, and 35 cm above the bottom of the terrarium). Three of these holes were located below the soil surface. Gas measurements were conducted by creating a cavity in the soil and inserting the gas sensor approximately 5 cm into it. In only a few cases, a tunnel was located next to a measurement hole, but no notable disparities in gas concentrations were detected which exceeded the natural fluctuations at all depths observed during the entire measurement period. After animals were placed in the terrarium, O_2_ concentrations were determined through the four holes to calculate mean O_2_ fraction over the past three consecutive days. During this period, the number of ventilation vents in the iron lid was optimized to achieve stable hypoxic conditions. Hypoxic conditions were defined as mean O_2_ fraction of the last three consecutive days ≤ 15%. Stable hypoxic conditions were achieved between 4 and 7 days (family one—7 days; family two—6 days; family three—4 days) after introducing the animals to the terrarium enabling them to acclimate to the new housing conditions. In total, the animals were kept under hypoxic conditions for seven days. The CO_2_ concentration automatically increased in parallel with decreasing O_2_. We referred to this condition as intermittent chronic hypoxia/hypercapnia because the animals were rarely observed aboveground. The animals occasionally came to the surface to forage (observational data).

**Table 1:** Information on experimental animals

Family	Sex	Weight (g)	Age (months)
1	Female	109.1	67
	Male	68.3	10
	Female	65.8	16
	Male	70.7	10
2	Male	86.0	56
	Male	70.1	13
	Female	64.6	49
3	Male	58.2	12
	Male	114.5	17
	Female	68.7	17
	Male	97.3	17
	Male	118.7	117

### Blood sampling and full blood count

Before and immediately after hypoxia treatment, blood samples were obtained by puncturing the vena saphena on the forepaw under ketamine (6 mg/kg) and xylazine (2.5 mg/kg) anesthesia (Garcia Montero et al. 2015). Samplings were conducted between 12 and 3 pm. Full blood was collected in a tube containing EDTA for a complete blood count using a fully automated hemocytometer (Vet abc, scil animal care company GmbH, Viernheim, Germany). The remaining blood was centrifuged at 3,500 rpm at room temperature to collect the serum for hormone measurements. Serum aliquots were stored at −80 °C until use.

### Thyroid hormone measurement

Serum concentrations of the fT3, TT4, and TT3 were determined with commercial ELISA kits according to manufacturer’s instructions (DRG Diagnostics, Marburg, Germany: fT3—EIA 2385; TT4—EIA 4568; TT3—EIA 4569). Detection limits were 0.536 pg/mL (fT3) 8 nmol/L (TT4), and 0.1 ng/mL (TT3). The specificity of the ELISA kits for mole-rat serum was validated previously (Gerhardt et al. 2023). TT4 concentrations of five samples were not detectable with the ELISA kit, because concentrations were too low. These samples were set to the detection limit of 8 nmol/L for statistical analysis.

### Statistical analysis

Statistical analyses were performed using GraphPad Prism (version 9.3.1, San Diego, CA, USA). All datasets were tested for normal distribution using Anderson-Darling, D’Agostino-Pearson Omnibus, and Shapiro Wilk test. Data were log-transformed when they were not normally distributed. Normal distributed data were analyzed using paired t-tests to compare pre- and post-treatment data. In case normal distribution was not achieved by log-transformation, Wilcoxon’s tests were applied. All data are expressed as mean ± SD. Statistical significance was defined as *p < 0.05 and **p < 0.01.

## Results

### Ansell’s mole-rats voluntarily chose hypoxic/hypercapnic burrows

**Fig. 1:**
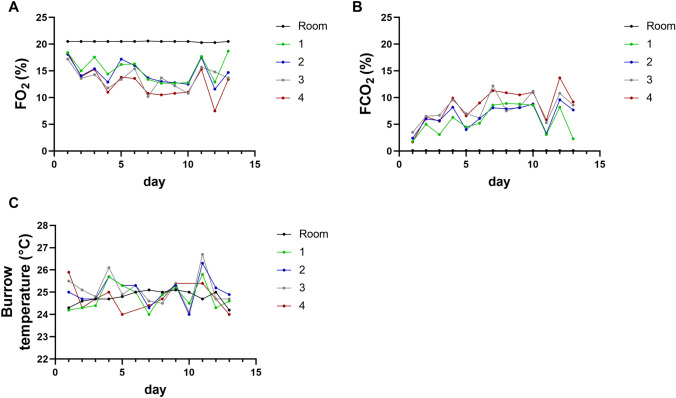
Representative graphs of atmospheric conditions in the hypoxia chamber. **A** O_2_ fractions (FO_2_), **B** CO_2_ fractions (FCO_2_), and **C** temperatures (°C) measured in the room air, the surface of the hypoxia chamber (hole 1; green), and three holes located below the soil surface (holes 2–4; blue, grey, red) during one treatment period of 13 days

Our aim was to keep the animals under chronic intermittent hypoxia and hypercapnia in self-constructed burrow systems to simulate natural burrow conditions. The animals started digging burrows immediately after being introduced to the experimental terrarium. Burrows were found at all levels of the terrarium. The animals were rarely observed aboveground, which was almost exclusively for foraging purposes, although O_2_ concentrations were the highest and CO_2_ concentrations were the lowest at the surface and the lid was equipped with vents. We measured O_2_ and CO_2_ concentrations at surface level and through the holes located below surface. The mean surface O_2_ and CO_2_ fractions measured in all three groups were 16.7 ± 1.3% (range 12.5–20.5%) and 4.4 ± 1.3% (range 0.2–9%), respectively. Mean subterranean O_2_ and CO_2_ fractions measured in all three groups (calculated over the measurements made in the three holes below the soil) were 14.9 ± 1.3% (range 7.5–20.5%) and 6.3 ± 1.4% (range 0.1–13.7%), respectively (Fig. 1A, B). Mean surface temperature measured in all groups was 25.2 ± 0.8 °C (range 22.5–27.5 °C). Mean subterranean temperature measured in all groups was 25.7 ± 0.7 °C (range 24.0–27.5 °C) (Fig. 1C).

### Serum fT3 was downregulated after chronic intermittent hypoxia/hypercapnia

**Fig. 2:**
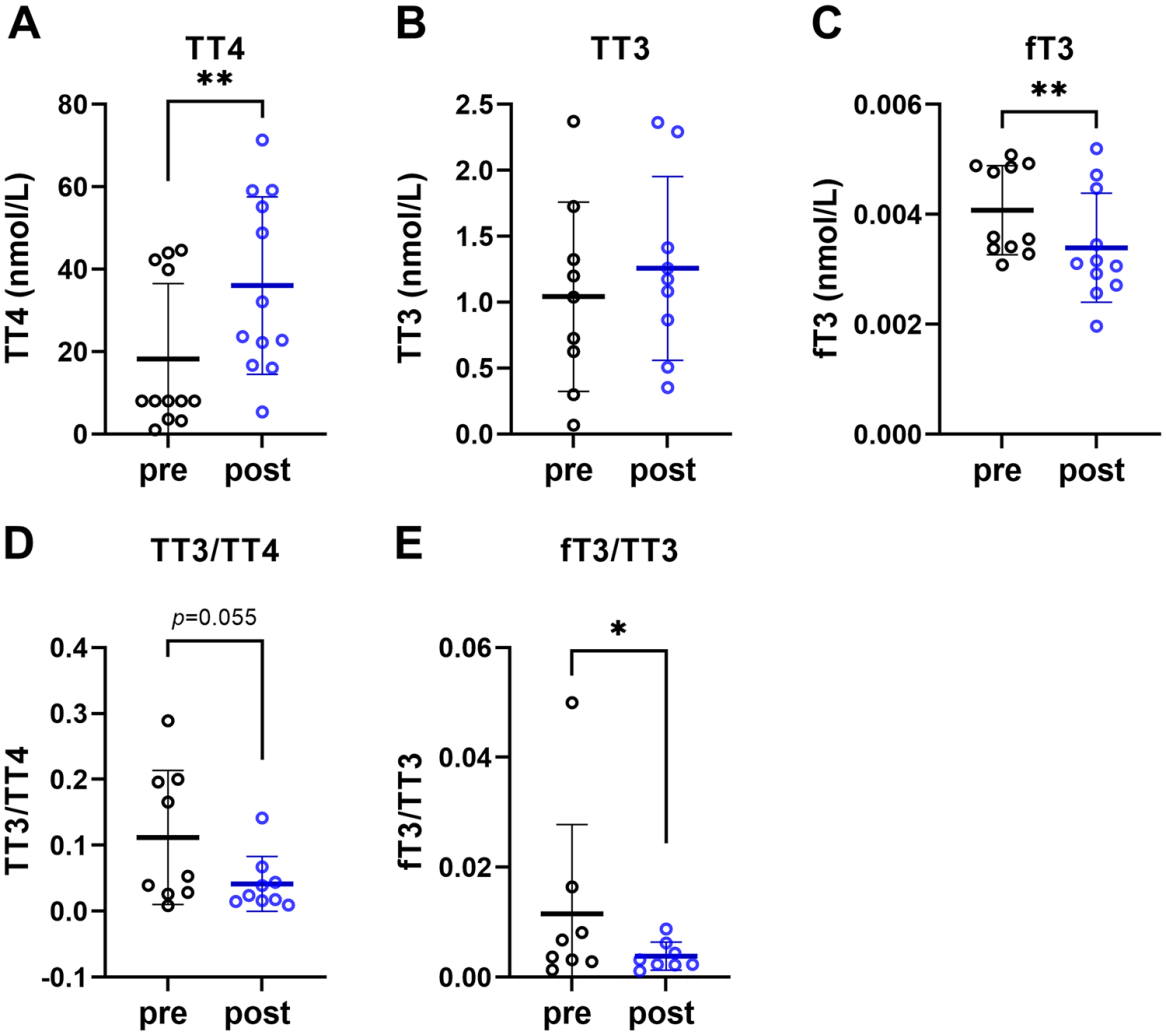
Serum thyroid hormone concentrations of Ansell’s mole-rats. **A** TT4, **B** TT3, **C** fT3 concentrations were measured in serum of Ansell’s mole-rats before (pre) and after (post) treatment under hypoxic/hypercapnic conditions using ELISA. Based on these concentrations, we determined **D** TT3/TT4 ratio as a measure for T3 to T4 conversion rates and **E** fT3/TT3 ratio as a measure for T3 activation. Hormone concentrations were statistically analyzed with paired t-tests or Wilcoxon tests depending on normal distribution (*N* = 9–12). Mean ± SD and all data points are depicted in the graphs. *p < 0.05 and **p < 0.01

TT4 concentrations were significantly higher after hypoxia/hypercapnia (Fig. 2A), however, there was no significant influence on TT3 (Fig. 2B). In contrast, fT3 concentrations were significantly lower after treatment (Fig. 2C). Furthermore, we calculated the ratios between TT3 and TT4 as well as fT3 and TT3 as a measure for T4 to T3 conversion rates and the recruitment of TT3 into its active form fT3, respectively. Although TT3 to TT4 ratios showed only a trend towards lower values after hypoxia/hypercapnia (*p* = 0.055; Fig. 2D), fT3 to TT3 ratios were significantly decreased after treatment (Fig. 2E).

### Erythropoiesis was downregulated after chronic intermittent hypoxia/hypercapnia

**Fig. 3:**
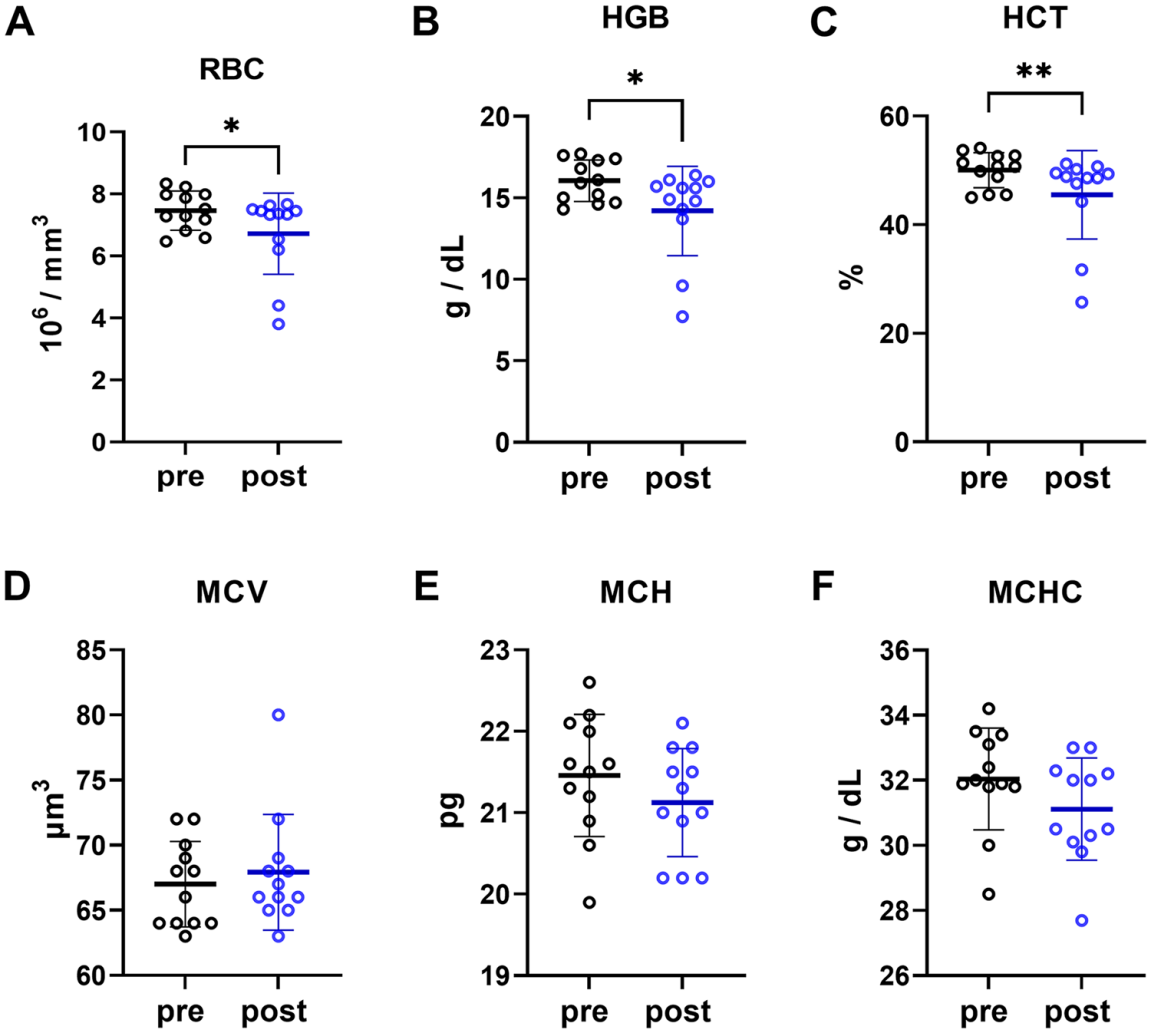
Hematological parameters of Ansell’s mole-rats. Full blood count was performed on blood of Ansell’s mole-rats before (pre) and after (post) treatment under hypoxic/hypercapnic conditions to measure **A** red blood cells (RBC), **B** hemoglobin (HGB), **C** hematocrit (HCT), **D** mean corpuscular volume (MCV), **E** mean corpuscular hemoglobin (MCH), and **F** mean corpuscular hemoglobin concentration (MCHC). Blood parameters were statistically analyzed with paired t-tests or Wilcoxon tests depending on normal distribution (*N* = 12). Mean ± SD and all data points are depicted in the graphs. *p < 0.05 and **p < 0.01

We measured hematological parameters before and after hypoxia/hypercapnia to analyze whether O_2_ transport is modulated under subterranean burrow conditions. In contrast to our expectation, we found the number of red blood cells (RBC), hemoglobin (HGB), and hematocrit (HCT) to be significantly downregulated after hypoxia/hypercapnia (Fig. 3A–C), while mean corpuscular volume (MCV), mean corpuscular hemoglobin (MCH), and mean corpuscular hemoglobin concentration (MCHC) were not affected (Fig. 3D–F). These results point towards downregulation of erythropoiesis while O_2_ loading of single erythrocytes was not influenced.

## Discussion

In the present study, we treated Ansell’s mole-rats under semi-natural conditions by providing housing conditions that enabled the animals to build underground burrows. The burrow atmosphere was hypoxic and hypercapnic after a few days, which enabled us to study long-term changes in serum TH concentrations and hematological parameters under chronic intermittent hypoxia/hypercapnia. As expected, when Ansell’s mole-rats are given the opportunity to dig, they will live underground despite the less favorable gas composition, emphasizing that these animals are well-adapted to hypoxic and hypercapnic conditions. The analysis of serum TH concentrations revealed that the animals had lower fT3 concentrations after hypoxia/hypercapnia treatment. Because fT3 is the transcriptionally-active fraction that can enter target cells, these findings indicate that TH signaling was downregulated at cellular level. Because THs are positive regulators of metabolic rate and thermogenesis (Mullur et al. 2014), our observations fit well with the studies that observed downregulation of RMR and *T*_b_ under hypoxic conditions in bathyergid mole-rats (Ivy et al. 2020; Pamenter et al. 2019; Farhat et al. 2020). Moreover, in four social African mole-rat species, it has been shown that hypoxia leads to a downregulation of uncoupling protein 1 (UCP1) in brown adipose tissue (BAT) (Cheng et al. 2021b). The UCP1 gene is a T3 target gene (Cannon and Nedergaard 2004; de Jesus et al. 2001), suggesting that downregulation of fT3 could contribute to hypoxia-driven downregulation of BAT thermogenesis. In contrast to fT3, we found that TT4 was upregulated by hypoxia/hypercapnia and TT3 was unaffected. Moreover, TT4 and TT3 concentrations showed high standard deviations, which could be ascribed to the individuals of family one (Table 1) that had higher hormone concentrations as compared to the other families. The changes in TH parameters after hypoxia/hypercapnia treatment allow us to infer the mechanism behind the downregulation of fT3, because T3 availability is mainly regulated by conversion of T4 into T3 within target cells by deiodinases types 1 and 2 (DIO1, DIO2) (van der Spek et al. 2017). As such, a lower TT3/TT4 ratio as observed in the post-treatment samples indicates either a downregulation of T4 to T3 conversion or higher T4 synthesis. Since we observed upregulated TT4 combined with unchanged TT3 concentrations, the most parsimonious explanation for the lower TT3/TT4 ratio is higher T4 synthesis in the thyroid, presumably triggered by decreased fT3 serum concentrations that activated the hypothalamus-pituitary-thyroid (HPT) axis via a negative feedback loop. However, due to the lower T4 to T3 conversion rates as well as a lower fraction of TT3 that is released into the free, transcriptionally-active form, indicated by the fT3/TT3 ratio, TH signaling seems to be ultimately downregulated in the periphery despite higher TT4. Interestingly, DIO3, which exclusively inactivates TH within cells, is activated under hypoxic conditions in several cell types including hepatocytes, neuronal cells, and cardiomyocytes (Simonides et al. 2008), proposing another cellular mechanism that could be utilized by mole-rats to further downregulate TH action at cellular level. However, to investigate regulatory effects via deiodinases, further experiments at the cellular level are required. Taken together, the downregulation of fT3 serum concentrations after hypoxia/hypercapnia is likely to be regulated by peripheral mechanisms, especially higher binding of T3 to plasma transport proteins such as albumin (Larsson et al. 1985; Davies 1993), possibly to downregulate RMR and *T*_b_. However, we must consider that the slightly higher temperature in the hypoxia chamber of on average 1 °C as compared to the mean temperature in the animal facilities could have contributed to downregulation of fT3 to avoid overheating. On the other hand, it has been shown that responses of the TH system to changes in ambient temperatures are under hypothalamic control, which regulates facultative thermogenesis upon changes in ambient temperature via modulating the HPT axis and autonomic responses (Martelli et al. 2014; Moffett et al. 2013; Tan et al. 2016). Therefore, it is unlikely that downregulation of serum fT3 concentrations is a response to slightly increased ambient temperatures in the hypoxia chambers, because TT4 and TT3 as a measure for TH synthesis were not downregulated after hypoxia/hypercapnia and even upregulated in case of TT4. Moreover, in naked mole-rats, it has been shown that the HPT axis responds to chronic cold exposure by increasing T4 synthesis in the thyroid (Buffenstein et al. 2001), supporting our conclusion that the observed peripheral changes in TH concentrations in the present study are not temperature-driven. As hypoxia alone is sufficient to downregulate RMR and *T*_b_ in African mole-rats (Farhat et al. 2020; Cheng et al. 2021b; Devereaux et al. 2023; Ivy et al. 2020), we infer from our data that specifically hypoxia has led to the observed changes in serum TH concentrations, representing the major upstream regulator of RMR and *T*_b_.

In the present study, we also analyzed changes in hematological parameters upon chronic intermittent hypoxia/hypercapnia treatment. Usually, hypoxia triggers erythropoiesis by activation of hypoxia-inducible factors (HIFs), which stimulate renal expression of the EPO gene and consequently erythropoiesis also in subterranean rodents (Jelkmann 2011; Shams et al. 2004; Li et al. 2021a, b). However, species adapted to hypoxic conditions do not always increase erythropoiesis and have evolved also alternative strategies to cope with low oxygen availability (Liu et al. 2010; Broekman et al. 2006; Pamenter 2022; Li et al. 2021a). Even in humans, adaptation to high altitudes does not necessarily involve upregulation of erythropoiesis (Beall 2000). In the present study, we found that all parameters associated with the number of erythrocytes (RBC, HGB, HCT) were downregulated upon chronic intermittent hypoxia/hypercapnia, while all parameters associated with erythrocyte size or HGB loading was not altered by treatment. These results point towards a downregulation of erythropoiesis with HGB concentrations remaining unchanged per erythrocyte but lower overall. In the first place, downregulation of oxygen transport capacities seems to be counterproductive under hypoxic conditions. However, African mole-rats downregulate energy turnover under hypoxic conditions (Ivy et al. 2020) and the reliance on anaerobic glucose metabolism to generate ATP might explain this contradiction (Ivy et al. 2020; Farhat et al. 2020; Pamenter et al. 2019). Furthermore, African mole-rats were reported to have highly efficient pH buffering capacities and seem to convert lactate, an acidic byproduct of anaerobic glycolysis, to glucose, allowing the animals to mitigate detrimental side effects of anaerobic metabolism (Ivy et al. 2020; Pamenter et al. 2019; Park et al. 2017). This is also in line with downregulation of serum fT3 concentrations under hypoxia because THs stimulate mitochondrial respiration (Mullur et al. 2014).

Taken together, we can infer from the present study that circulating fT3 serves as an upstream regulator of metabolic and thermoregulatory adaptations to hypoxia. Furthermore, lower hematocrit levels are in line with the suppressed energy demand and the switch to anaerobic metabolism under hypoxia.

## Data Availability

Data generated and analyzed during the current study will be made available upon request.
